# Inhibition of matrix metalloproteinases by HIV-1 integrase strand transfer inhibitors

**DOI:** 10.3389/ftox.2023.1113032

**Published:** 2023-02-21

**Authors:** Emma G. Foster, Nicholas Y. Palermo, Yutong Liu, Benson Edagwa, Howard E. Gendelman, Aditya N. Bade

**Affiliations:** ^1^ Department of Pharmacology and Experimental Neuroscience, University of Nebraska Medical Center, Omaha, NE, United States; ^2^ Computational Chemistry Core, University of Nebraska Medical Center, Omaha, NE, United States; ^3^ Department of Radiology, University of Nebraska Medical Center, Omaha, NE, United States; ^4^ Department of Pharmaceutical Sciences, University of Nebraska Medical Center, Omaha, NeE, United States

**Keywords:** HIV-1, pregnancy, antiretroviral drugs, integrase strand transfer inhibitors, neurodevelopment, drug-induced adverse events

## Abstract

More than fifteen million women with the human immunodeficiency virus type-1 (HIV-1) infection are of childbearing age world-wide. Due to improved and affordable access to antiretroviral therapy (ART), the number of *in utero* antiretroviral drug (ARV)-exposed children has exceeded a million and continues to grow. While most recommended ART taken during pregnancy suppresses mother to child viral transmission, the knowledge of drug safety linked to fetal neurodevelopment remains an area of active investigation. For example, few studies have suggested that ARV use can be associated with neural tube defects (NTDs) and most notably with the integrase strand transfer inhibitor (INSTI) dolutegravir (DTG). After risk benefit assessments, the World Health Organization (WHO) made recommendations for DTG usage as a first and second-line preferred treatment for infected populations including pregnant women and those of childbearing age. Nonetheless, long-term safety concerns remain for fetal health. This has led to a number of recent studies underscoring the need for biomarkers to elucidate potential mechanisms underlying long-term neurodevelopmental adverse events. With this goal in mind, we now report the inhibition of matrix metalloproteinases (MMPs) activities by INSTIs as an ARV class effect. Balanced MMPs activities play a crucial role in fetal neurodevelopment. Inhibition of MMPs activities by INSTIs during neurodevelopment could be a potential mechanism for adverse events. Thus, comprehensive molecular docking testing of the INSTIs, DTG, bictegravir (BIC), and cabotegravir (CAB), against twenty-three human MMPs showed broad-spectrum inhibition. With a metal chelating chemical property, each of the INSTI were shown to bind Zn++ at the MMP’s catalytic domain leading to MMP inhibition but to variable binding energies. These results were validated in myeloid cell culture experiments demonstrating MMP-2 and 9 inhibitions by DTG, BIC and CAB and even at higher degree than doxycycline (DOX). Altogether, these data provide a potential mechanism for how INSTIs could affect fetal neurodevelopment.

## Introduction

Pregnant women and women of child bearing age infected with the human immunodeficiency virus type-1 (HIV-1) infection have benefited by antiretroviral therapy (ART) in the reduction of maternal fetal viral transmission (The U.S. Department of Health and Human Services, 2015; World Health Organization (WHO), 2019a). Currently, more than 15.5 million women of child-bearing age are HIV-1 infected, worldwide (The Joint United Nations Programme on HIV/AIDS (UNAIDS), 2021a). In 2020, eighty five percent of HIV-1-infected pregnant women were on ART (The Joint United Nations Programme on HIV/AIDS (UNAIDS), 2021a). Due to such broad usage of ART during pregnancy, the rate of vertical transmission of HIV-1 has reduced to less than 1% (The Centers for Disease Control and Prevention (CDC), 2018; Peters et al., 2017; Schnoll et al., 2019; Rasi et al., 2022; The Joint United Nations Programme on HIV/AIDS (UNAIDS), 2021b). This includes resource-limited countries (RLCs), which currently hold up to two-thirds of the world’s total HIV-1 infected population (The Joint United Nations Programme on HIV/AIDS (UNAIDS), 2021b). However, along with the significant benefits in reducing infection-associated morbidities and mortalities, there remains risks of ART-linked adverse events (Hill et al., 2018). As over a million ARV-exposed HIV-1 uninfected children are born each year (Ramokolo et al., 2019; Crowell et al., 2020), an appreciation of adverse pregnancy events, in particular, related to ARVs is certainly warranted.

Herein, we particularly focused on HIV-1 integrase strand transfer inhibitors (INSTIs), a relatively new class of ARVs. Raltegravir (RAL), elvitegravir (EVG), dolutegravir (DTG), bictegravir (BIC), and cabotegravir (CAB) are the US Food and Drug Administration (FDA) approved INSTIs for the treatment of HIV-1 infected patients (Smith et al., 2021). In recent years, widespread usage of INSTIs have emerged related to their efficacy and high barrier to viral drug resistance (Smith et al., 2021). Indeed, these antiretrovirals are currently part of preferred first- and second-line ART regimens (World Health Organization (WHO), 2016; Department of Health and Human Services (DHHS), 2022). Moreover, increasing pretreatment resistance to non-nucleoside reverse transcriptase inhibitors (NNRTIs) in RLCs, especially in women, increases usage of INSTI-based regimens (World Health Organization (WHO), 2019a; World Health Organization (WHO), 2019b). During pregnancy, DTG and RAL are preferred drugs in combination therapy with a preferred dual-nucleoside reverse transcriptase inhibitor (NRTI) backbone. EVG, BIC or CAB are not recommended during pregnancy due to limited safety data (The U.S. Department of Health and Human Services, 2015). Recently DTG was found to be potentially associated with birth defects (NTDs) and postnatal neurodevelopmental abnormalities (Hill et al., 2018; Cabrera et al., 2019; Zash et al., 2019; Crowell et al., 2020; Mohan et al., 2020; Bade et al., 2021). Given the widescale usage of DTG as a part of first-line regimens worldwide (Hill et al., 2018; Dorward et al., 2018; World Health Organization (WHO), 2018; The Lancet, 2020) and emerging potent INSTIs such as BIC and CAB, uncovering any INSTIs-associated adverse effects and thus, the underlying mechanisms is of importance.

Pre-clinical and clinical research have served to evaluate interaction between folate levels or transport pathways and DTG or other INSTIs for any developmental toxicity (Cabrera et al., 2019; Chandiwana et al., 2020; Gilmore et al., 2022). However, results have failed to conclusively establish cause-and-effect relationships (Cabrera et al., 2019; Chandiwana et al., 2020; Gilmore et al., 2022). No other biomarker linked to INSTI drug-induced adverse events has been explored. We demonstrated that DTG is a broad-spectrum inhibitor of matrix metalloproteinases (MMPs) (Bade et al., 2021). MMPs are known to play a role in many neurodevelopmental processes, including, but not limited to axonal growth and guidance, synaptic development and plasticity (Ethell and Ethell, 2007; Agrawal et al., 2008; Fujioka et al., 2012; Reinhard et al., 2015; De Stefano and Herrero, 2017). Therefore, dysregulation of their activities could affect fetal neurodevelopment (Reinhard et al., 2015; Bade et al., 2021). Docking assessments against five MMPs showed that DTG binds to Zn++ at the catalytic domain of an MMP to inhibit the enzyme’s activity. Moreover, such MMPs inhibition can affect mice fetal neurodevelopment following DTG administration to pregnant dams at the time of conception. Clinical reports of adverse events associated with INSTIs have demonstrated class effects. Therefore, it is prudent to determine whether other ARVs from the INSTI class are inhibitors of MMPs and consider this as a potential mechanism of INSTIs-related adverse neurodevelopmental outcomes. Moreover, such biomarker discovery against MMP enzymes will help to understand potential genetic susceptibility. Herein, we show, for the first time, comprehensive computational molecular docking assessments of DTG, BIC or CAB against each one of the twenty-three human MMP enzymes. Further, inhibition potency of each INSTI was validated using a cell culture model. To this end, we show that inhibition of MMPs activities is an INSTI class effect and warranting assessments to determine the effect of drug-induced effects on the gestational environment and fetal neurodevelopment.

## Methods

### Molecular docking

Homology models of all 23 known human MMPs (MMP-1, 2, 3, 7, 8, 9, 10, 11, 12, 13, 14, 15, 16, 17, 19, 20, 21, 23, 24, 25, 26, 27, and 28) were generated. This was done on a template of MMP-2 (PDB ID: 1HOV) using the Homology Modeling module of the YASARA Structure program package (Krieger and Vriend, 2014). The Schrodinger software suite release 2020–4 (New York, NY) was used for all molecular dynamic simulations and molecular docking calculations. All molecules were parametrized using the OPLS3e force field (Harder et al., 2016). Each homology model was placed in an orthorhombic box of TIP4P water with periodic boundaries; at least 10 Å from any solute molecule. The simulation cells were neutralized with the addition of Na + or Cl− ions. Production molecular dynamics were run for 500 ns with default settings. The representative structure of the largest cluster from each simulation was chosen for docking calculations. Induced-fit binding as implemented in Schrodinger was used with default settings, except that the high-accuracy XP mode was chosen for Glide docking. All ranked poses were required to have at least one bond with the active site zinc ion; other poses were not considered.

### Gelatin zymography

Gelatin zymography was performed to assess MMP-9 and -2 activity following treatment of THP-1 cells with DTG, CAB, BIC, or DOX. This assay was used as preliminary confirmation of the inhibition of MMPs by individual INSTIs. Due to the nature of this assay, only the gelatinases, MMP-2 and -9, could be assessed. Cells were plated at a density of 1 × 10^6^ in 12 well plates and treated with phorbol-12-myristate-13-acetate (PMA) for 24 h. This was done to promote cell differentiation to stimulate MMP secretion. Following PMA treatment, cells were treated with DTG, CAB, BIC, or DOX at concentrations of 25, 50, 75, or 100 µM or control vehicle for 24 h. In our previous study, no DTG-induced cytotoxicity was recorded in PMA-stimulated THP-1 cells up to 100 µM (Bade et al., 2021). Thus, for comparative assessments among different INSTIs (DTG, BIC, and CAB) and DOX (positive control) drug concentrations of up to 100 µM were utilized. Each of the experimental tests were performed in triplicate. Following treatment, media was collected and centrifuged at 15,000 x g for 10 min at 4°C. Supernatant was collected and stored at −80°C for further analysis. For gelatin zymography, 3 µg of protein from cell medium was loaded in a 10% SDS-polyacrylamide gel containing 0.1% gelatin. Gels were ran at 55 V until the loading dye passed through its bottom. The gel was then removed and washed with water for 15 min, then incubated with renaturation buffer [2.5% (v/v) Triton X-100 in Milli-Q water] for 90 min at room temperature. The used renaturation buffer was replaced with fresh buffer every 30 min. Renaturation buffer was then replaced with developing buffer (50 mM Tris–HCl, pH 7.5, 5 mM CaCl2, 0.2 M NaCl, and 0.02% Brij-35) and the gel was incubated at 37°C in a shaker (Innova 42, New Brunswick Scientifc, Edison, NJ) for 48 h. After 48 h, the gel was washed with water for 15 min and then stained using 0.2% Coomassie Brilliant Blue R-250 (BIO-RAD, Hercules, CA) for 1 h. After staining, the gel was washed with water for 15 min before washing with destaining solution (30% methanol, 10% acetic acid, 60% water) for 45 min. The gel was then washed with water for 20 min to remove any destaining solution. Finally, the stained gel was imaged using the iBright 750 Imaging System (Invitrogen, Carlsbad, CA). ImageJ software was used to quantitate band density recorded as a measure of relative MMP activity.

### Statistical analysis

Statistical analyses were conducted using GraphPad Prism 7.0 software (La Jolla, CA). Data from *in vitro* studies were expressed as mean ± standard error of the mean (SEM) with a minimum of 3 biological replicates. A one-way ANOVA followed by Tukey’s or Dunnett’s test was used to compare three or more groups. Statistical significance was denoted as *p < 0.05, **p < 0.01, ***p < 0.001, ****p < 0.0001.

## Results

### INSTIs chelate Zn++ at the catalytic domain of MMPs

Molecular dockings were completed using Schrodinger’s software to identify the mechanism through which each INSTI interacts with the catalytic domain structures of the human MMPs. Here, we used DTG, BIC and CAB for assessments. MMPs are Zn++ dependent endopeptidases. INSTIs possess a prominent metal-binding pharmacophore (MBP) also, referred to as a metal-binding group or MBG in their chemical structure. Based on these chemical abilities for binding to the metal ions we hypothesized that DTG, BIC or CAB can inhibit MMPs activities by binding to Zn++ at the catalytic domain of the protein structure. Herein, induced fit docking used a combination of the Glide and Prime programs in the Schrodinger suite. All docking scores used the highest accuracy Glide XP mode. Previously, we reported interaction of DTG with five MMPs, MMP-2, 8, 9, 14, and 19 and interaction of CAB or BIC with MMP-2 and -14 as proof-of-concept evaluations (Bade et al., 2021). Herein, as 23 MMPs are known to be found in humans, molecular docking interaction was tested against each of these enzymes to find the highest binding interaction for DTG, BIC or CAB and determine whether any individual MMP could have genetic susceptibility against these INSTIs.

DTG formed a metal coordination complex with Zn^++.^ This was recorded in the catalytic domain of each of the MMPs tested. Metal coordination of DTG with Zn++ occurred at Zn 166, 166, 479, 269, 469, 709, 478, 490, 472, 473, 584, 671, 609, 605, 510, 485, 571, 485, 647, 564, 263, 515, and 522 receptors of MMP-1, 2, 3, 7, 8, 9, 10, 11, 12, 13, 14, 15, 16, 17, 19, 20, 21, 23, 24, 25, 26, 27, and 28, respectively. DTG also formed other interactions with Zn^++^, which included cation pi interactions. These interactions occurred at Zn 490, 605, and 522 receptors of MMP-11, 17, and 28 respectively. Other interactions included pi stacking and hydrogen bonding. Pi stacking occurred with histidine amino acids of all tested MMPs except MMP-7, 9, 19, and 27. Pi stacking also occurred with tyrosine amino acids, but only with MMP-3, 11, and 16. Hydrogen bond interactions occurred at glutamate amino acid residues of MMP-1, 3, 10, 12, 15, 16, 19, 20, 21, 23, 24, 25, and 26; alanine amino acid residues of MMP-2, 7, 8, 11, 14, 17, and 19; leucine amino acid residues of MMP-7, 8, 9, 10, 12, 15, 16, 20, 23, 24, 25, and 26; glycine amino acid residues of MMP-9 and 27; tyrosine amino acid residues of MMP-9 and 27; asparagine 170 amino acid residue of MMP-8; proline 421 amino acid residue of MMP-9; phenylalanine 249 of MMP-21; and glutamine 247 of MMP-21. The distances of all the receptor-ligand bonds are shown in the respective DTG-MMP interaction table (Table 1). Docking simulation of DTG into individual MMP showed binding energy of −6.032, −6.450, −6.253, −7.243, −8.330, −9.430, −6.686, −6.210, −6.713, −6.461, −9.040, −5.885, −7.325, −6.810, −7.130, −6.097, −6.179, −6.213, −6.917, −6.865, −7.198, −7.427 or −6.012 kcal/mol for MMP-1 to −28, respectively (Table 4). Overall, observed high binding energies from the docking simulation validated docking interactions in Table 1. Moreover, these docking assessments confirmed that DTG is a broad-spectrum inhibitor, and it inhibits all MMPs activities by chelating Zn++ at the catalytic domain.

**TABLE 1:** Dolutegravir (DTG)- Matrix metalloproteinases (MMPs) Interactions.

MMP-1 interactions			
Ligand	Receptor	Type	Distance (Å)
Ar1	His 218	Pi stacking	3.60
NH	Glu 219	Hydrogen bond	1.98
O2	Zn 166	Metal coordination	2.01

MMP-2 interactions			
Ligand	Receptor	Type	Distance (Å)
Ar1	His 120	Pi stacking	3.59
NH	Ala 84	Hydrogen bond	2.28
O1	Zn 166	Metal coordination	2.19
O2	Zn 166	Metal coordination	2.21

MMP-3 interactions			
Ligand	Receptor	Type	Distance (Å)
Ar1	His 218	Pi stacking	3.57
Ar1	Tyr 240	Pi stacking	5.32
NH	Glu 219	Hydrogen bond	2.36
O2	Zn 479	Metal coordination	2.13

MMP-7 interactions			
Ligand	Receptor	Type	Distance (Å)
O1	Leu 176	Hydrogen bond	2.02
O1	Ala 177	Hydrogen bond	2.07
O2	Zn 269	Metal coordination	2.14

MMP-8 interactions			
Ligand	Receptor	Type	Distance (Å)
Ar1	His 217	Pi stacking	3.8
O1	Leu 180	Hydrogen bond	1.93
O1	Ala 181	Hydrogen bond	2.32
O2	Zn 469	Metal coordination	2.02
O5	Asn 170	Hydrogen bond	1.91

MMP-9 interactions			
Ligand	Receptor	Type	Distance (Å)
NH	Gly 186	Hydrogen bond	1.87
O1	Try 423	Hydrogen bond	1.96
O3	Pro 421	Hydrogen bond	2.09
O4	Zn 709	Metal coordination	2.14
O5	Leu 188	Hydrogen bond	2.80

MMP-10 interactions			
Ligand	Receptor	Type	Distance (Å)
Ar1	His 217	Pi stacking	3.42
NH	Glu 218	Hydrogen bond	1.99
O1	Leu 180	Hydrogen bond	2.67
O2	Zn 478	Metal coordination	2.15

MMP-11 interactions			
Ligand	Receptor	Type	Distance (Å)
Ar1	His 215	Pi stacking	4.03
Ar1	Tyr 237	Pi Stacking	5.48
Ar1	Zn 490	Cation pi	4.63
NH	Ala 178	Hydrogen bond	1.91
O2	Zn 490	Metal coordination	2.13

MMP-12 interactions			
Ligand	Receptor	Type	Distance (Å)
Ar1	His 218	Pi stacking	3.75
NH	Glu 219	Hydrogen bond	2.22
O1	Leu 181	Hydrogen bond	2.1
O2	Zn 472	Metal coordination	2.13

MMP-13 interactions			
Ligand	Receptor	Type	Distance (Å)
Ar1	His 222	Pi stacking	3.72
O2	Zn 473	Metal coordination	2.07

MMP-14 interactions			
Ligand	Receptor	Type	Distance (Å)
Ar2	His 239	Pi stacking	3.49
O3	Ala 258	Hydrogen bond	1.87
O4	Zn 584	Metal coordination	2.12

MMP-15 interactions			
Ligand	Receptor	Type	Distance (Å)
Ar1	His 259	Pi stacking	3.4
NH	Glu 260	Hydrogen bond	2.03
O1	Leu 219	Hydrogen bond	2.22
O2	Zn 671	Metal coordination	2.06

MMP-16 interactions			
Ligand	Receptor	Type	Distance (Å)
Ar1	His 246	Pi stacking	3.34
Ar1	Tyr 268	Pi Stacking	5.43
NH	Glu 247	Hydrogen bond	2.11
O1	Leu 206	Hydrogen bond	2.07
O2	Zn 609	Metal coordination	2.05

MMP-17 interactions			
Ligand	Receptor	Type	Distance (Å)
Ar1	His 259	Pi stacking	3.89
Ar1	Zn 605	Cation pi	4.72
NH	Ala 208	Hydrogen bond	1.89
O2	Zn 605	Metal coordination	2.09

MMP-19 interactions			
Ligand	Receptor	Type	Distance (Å)
O1	Zn 510	Metal coordination	2.10
O3	Ala 231	Hydrogen bond	2.03
O4	Glu 235	Hydrogen bond	2.14

MMP-20 interactions			
Ligand	Receptor	Type	Distance (Å)
Ar1	His 226	Pi stacking	3.49
NH	Glu 227	Hydrogen bond	2.06
O1	Leu 189	Hydrogen bond	2.26
O2	Zn 485	Metal coordination	2.05

MMP-21 interactions			
Ligand	Receptor	Type	Distance (Å)
Ar1	His 283	Pi stacking	3.52
NH	Glu 284	Hydrogen bond	2.00
O1	Phe 249	Hydrogen bond	2.03
O2	Zn 571	Metal coordination	2.11
O5	Gln 247	Hydrogen bond	2.60

MMP-23 interactions			
Ligand	Receptor	Type	Distance (Å)
Ar1	His 226	Pi stacking	3.59
NH	Glu 227	Hydrogen bond	2.18
O1	Leu 189	Hydrogen bond	2.23
O2	Zn 485	Metal coordination	2.09

MMP-24 interactions			
Ligand	Receptor	Type	Distance (Å)
Ar1	His 282	Pi stacking	3.44
NH	Glu 283	Hydrogen bond	2.19
O1	Leu 242	Hydrogen bond	2.20
O2	Zn 647	Metal coordination	2.09

MMP-25 interactions			
Ligand	Receptor	Type	Distance (Å)
Ar1	His 233	Pi stacking	3.43
NH	Glu 234	Hydrogen bond	2.63
O1	Leu 192	Hydrogen bond	2.06
O2	Zn 564	Metal coordination	2.02

MMP-26 interactions			
Ligand	Receptor	Type	Distance (Å)
Ar1	His 208	Pi stacking	3.41
NH	Glu 209	Hydrogen bond	2.6
O1	Leu 171	Hydrogen bond	2.46
O2	Zn 263	Metal coordination	2.09

MMP-27 interactions			
Ligand	Receptor	Type	Distance (Å)
NH	Gly 177	Hydrogen bond	2.08
O1	Tyr 238	Hydrogen bond	1.79
O3	Zn 515	Metal coordination	2.22
O4	Zn 515	Metal coordination	2.27

MMP-28 interactions			
Ligand	Receptor	Type	Distance (Å)
Ar1	His 240	Pi stacking	3.87
Ar1	Zn 522	Cation pi	4.75
O2	Zn 522	Metal coordination	2.12

Notably, CAB also formed a metal coordination complex with Zn^++^ in the catalytic domain of all tested MMPs. Metal coordination of CAB with Zn++ occurred at Zn 471, 166, 479, 269, 469, 709, 478, 490, 472, 473, 584, 671, 609, 605, 510, 485, 571, 392, 647, 564, 263, 515, and 522 receptors of MMP-1, 2, 3, 7, 8, 9, 10, 11, 12, 13, 14, 15, 16, 17, 19, 20, 21, 23, 24, 25, 26, 27, and 28, respectively. However, CAB also formed salt bridges with Zn++ of all tested MMPs. In addition, salt bridge interactions occurred with glutamate amino acids of MMP-1, 8, 9, 10, 12, 14, 17, 19, 21, 23, 24, and 26. Salt bridges with Zn++ or with other amino acids were not observed with any of DTG-MMP interactions. Other interactions between CAB and MMPs were cation pi, pi stacking and hydrogen bonding. Cation pi interactions occurred at histidine 263 and phenylalanine 205 of MMP-15 and 16 respectively. Pi stacking interactions occurred with histidine amino acids of MMP-1, 2, 11, 12, 15, 17, 19, 21, 23, 25, and 28; phenylalanine amino acids of MMP-1 and 16; tyrosine amino acids of MMP-2, 14, and 19. Hydrogen bonding of CAB with tested MMPs was found, except MMP-17. Like DTG, CAB was found to produce hydrogen bonding with leucine, alanine, glutamate, phenylalanine, glycine, proline, asparagine, and tyrosine amino acid residues. However, other hydrogen bonding occurred at serine 239 amino acid residue of MMP-1, valine 233 amino acid residue of MMP-19, and arginine 240 amino acid residue of MMP-23. The distances of all the receptor-ligand bonds are shown in the respective CAB-MMP interaction table (Table 2). Docking simulation of CAB into individual MMP showed binding energy of −14.251, −8.588, −15.222, −12.305, −14.337, −14.222, −14.592, −10.352, −12.19, −11.798, −12.983 −9.632, −12.249, −11.666, −12.718, −12.413, −14.389, −13.109, −15.339, −11.62, −12.381, −12.614, or −10.11 kcal/mol for MMP-1 to −28, respectively (Table 4). Altogether, observed high binding energies from the docking simulation and docking interactions (Table 1; Table 4) evaluations confirmed that CAB is a broad-spectrum inhibitor, and it inhibits all MMPs activities by binding to Zn++ at the catalytic domain.

**TABLE 2:** Cabotegravir (CAB)- Matrix metalloproteinases (MMPs) Interactions.

MMP-1 Interactions			
Ligand	Receptor	Type	Distance (Å)
Ar1	His 218	Pi stacking	4.9
Ar1	Phe 242	Pi stacking	5.28
O2	Ser 239	Hydrogen bond	2.28
O2	Zn 471	Salt bridge	2.14
O3	Zn 471	Metal coordination	2.08
O3	Zn 471	Salt bridge	2.08
N2	Glu 219	Salt bridge	4.91

MMP-2 Interactions			
Ligand	Receptor	Type	Distance (Å)
Ar1	His 120	Pi stacking	3.34
Ar1	Tyr 142	Pi stacking	5.07
O1	Leu 83	Hydrogen bond	1.99
O1	Ala 84	Hydrogen bond	2.40
O2	Glu 121	Hydrogen bond	1.92
O2	Zn 166	Metal coordination	2.25
O3	Zn 166	Metal coordination	2.05
O3	Zn 166	Salt bridge	2.05

MMP-3 Interactions			
Ligand	Receptor	Type	Distance (Å)
O1	Leu 181	Hydrogen bond	1.76
N1	Pro 238	Hydrogen bond	1.96
O2	Zn 479	Metal coordination	2.41
O3	Zn 479	Metal coordination	2.00
O3	Zn 479	Salt bridge	2.00

MMP-7 Interactions			
Ligand	Receptor	Type	Distance (Å)
O1	Leu 176	Hydrogen bond	2.05
O1	Ala 177	Hydrogen bond	2.1
O2	Glu 215	Hydrogen bond	2.14
O2	Zn 269	Metal coordination	2.39
O3	Zn 269	Metal coordination	2.01
O3	Zn 269	Salt bridge	2.01

MMP-8 Interactions			
Ligand	Receptor	Type	Distance (Å)
O1	Asn 170	Hydrogen bond	2.03
O3	Zn 469	Metal coordination	2.14
O3	Zn 469	Salt bridge	2.14
N2	Glu 218	Salt bridge	4.78
O4	Zn 469	Metal coordination	2.05
O5	Leu 180	Hydrogen bond	1.76

MMP-9 Interactions			
Ligand	Receptor	Type	Distance (Å)
N1	Pro 421	Hydrogen bond	2.03
O1	Leu 188	Hydrogen bond	1.95
O2	Zn 709	Metal coordination	2.24
N2	Glu 402	Salt bridge	4.73
O3	Zn 709	Metal coordination	1.98
O3	Zn 709	Salt bridge	1.98

MMP-10 Interactions			
Ligand	Receptor	Type	Distance (Å)
N1	Pro 237	Hydrogen bond	2.24
O1	Leu 180	Hydrogen bond	1.95
O2	Zn 478	Metal coordination	2.37
O3	Zn 478	Metal coordination	1.92
O3	Zn 478	Salt bridge	1.92
N2	Glu 218	Salt bridge	4.77

MMP-11 Interactions			
Ligand	Receptor	Type	Distance (Å)
Ar1	His 215	Pi stacking	3.98
O1	Leu 177	Hydrogen bond	2.2
O2	Zn 490	Metal coordination	2.08
O3	Zn 490	Metal coordination	2.10
O3	Zn 490	Salt bridge	2.10

MMP-12 Interactions			
Ligand	Receptor	Type	Distance (Å)
Ar1	His 183	Pi stacking	4.16
O3	Zn 472	Metal coordination	2.05
O3	Zn 472	Salt bridge	2.05
N2	Glu 219	Salt bridge	4.27
O4	Zn 472	Metal coordination	2.23
O5	Leu 181	Hydroen bond	1.94

MMP-13 Interactions			
Ligand	Receptor	Type	Distance (Å)
N1	Pro 242	Hydrogen bond	2.11
O1	Leu 185	Hydrogen bond	2.11
O2	Zn 473	Metal coordination	2.09
O3	Zn 473	Metal coordination	1.92
O3	Zn 473	Salt bridge	1.92

MMP-14 Interactions			
Ligand	Receptor	Type	Distance (Å)
Ar1	His 239	Pi stacking	3.76
Ar1	Tyr 261	Pi stacking	5.36
Ar2	His 239	Pi stacking	4.98
O2	Zn 584	Metal coordination	2.33
O3	Zn 584	Metal coordination	2.05
O3	Zn 584	Salt bridge	2.05
O5	Ala 202	Hydrogen bond	2.42
N1	Glu 240	Hydrogen bond	2.15
N2	Glu 240	Salt bridge	3.15

MMP-15 Interactions			
Ligand	Receptor	Type	Distance (Å)
Ar1	Tyr 223	Pi stack	4.84
O1	Ala 222	Hydrogen bond	1.94
O2	Glu 260	Hydrogen bond	2.23
O3	Zn 671	Metal coordination	1.96
O3	Zn 671	Salt bridge	1.96
N2	His 263	Cation pi	5.84
O4	Zn 671	Metal coordination	2.29

MMP-16 Interactions			
Ligand	Receptor	Type	Distance (Å)
O1	Leu 206	Hydrogen bond	2.17
O1	Ala 207	Hydrogen bond	1.84
O2	Glu 247	Hydrogen bond	1.74
O2	Zn 609	Metal coordination	2.49
Ar2	Phe 205	Pi stacking	4.25
O3	Zn 609	Metal coordination	2.03
O3	Zn 609	Salt bridge	2.03
N2	Phe 205	Cation pi	3.97

MMP-17 Interactions			
Ligand	Receptor	Type	Distance (Å)
Ar1	His 248	Pi stacking	4.3
O2	Zn 605	Metal coordination	1.98
O3	Zn 605	Salt bridge	2.14
O3	Zn 605	Metal coordination	2.14
N2	Glu 249	Salt bridge	4.98

MMP-19 Interactions			
Ligand	Receptor	Type	Distance (Å)
O2	Val 233	Hydrogen bond	1.53
Ar2	His 212	Pi stacking	3.92
Ar2	Tyr 234	Pi stacking	4.27
O3	Zn 510	Metal coordination	2.13
O3	Zn 510	Salt bridge	2.13
N2	Glu 213	Salt bridge	3.36
O4	Zn 510	Metal coordination	2.42

MMP-20 Interactions			
Ligand	Receptor	Type	Distance (Å)
O1	Leu 189	Hydrogen bond	1.86
O1	Ala 190	Hydrogen bond	2.1
O2	Glu 227	Hydrogen bond	1.94
O2	Zn 485	Metal coordination	2.37
O3	Zn 485	Metal coordination	2.15
O3	Zn 485	Salt bridge	2.15

MMP-21 Interactions			
Ligand	Receptor	Type	Distance (Å)
Ar1	His 283	Pi stacking	4.76
N1	Glu 284	Hydrogen bond	1.69
O1	Phe 249	Hydrogen bond	2.1
O2	Zn 571	Metal coordination	2.11
O3	Zn 571	Metal coordination	2.15
O3	Zn 571	Salt bridge	2.15
N2	Glu 248	Salt bridge	4.83
O5	Arg 240	Hydrogen bond	1.87

MMP-23 Interactions			
Ligand	Receptor	Type	Distance (Å)
Ar1	His 211	Pi stacking	4.19
O1	Leu 168	Hydrogen bond	2.01
O2	Ala 169	Hydrogen bond	2.48
O2	Glu 212	Hydrogen bond	2.25
O3	Zn 392	Metal coordination	2
O3	Zn 392	Salt bridge	2
N2	Glu 167	Salt bridge	3.36

MMP-24 Interactions			
Ligand	Receptor	Type	Distance (Å)
O1	Leu 242	Hydrogen bond	2.14
O2	Zn 647	Metal coordination	2.28
O3	Zn 647	Salt bridge	2.12
O3	Zn 647	Metal coordination	2.12
N2	Glu 283	Salt bridge	4.63

MMP-25 Interactions			
Ligand	Receptor	Type	Distance (Å)
Ar1	His 233	Pi stacking	3.52
O1	Leu 192	Hydrogen bond	1.79
O1	Ala 193	Hydrogen bond	2.02
O2	Glu 234	Hydrogen bond	1.72
O2	Zn 564	Metal coordination	2.18
O3	Zn 564	Salt bridge	2.06
O3	Zn 564	Metal coordination	2.06

MMP-26 Interactions			
Ligand	Receptor	Type	Distance (Å)
O3	Zn 263	Metal coordination	1.99
O3	Zn 263	Salt bridge	1.99
N2	Glu 209	Salt bridge	4.9
O4	Zn 263	Metal coordination	2.22
O5	Leu 171	Hydrogen bond	1.92

MMP-27 Interactions			
Ligand	Receptor	Type	Distance (Å)
O1	Gly 180	Hydrogen bond	1.99
O2	Glu 217	Hydrogen bond	1.66
O2	Zn 515	Metal coordination	2.48
O3	Zn 515	Salt bridge	2.11
O3	Zn 515	Metal coordination	2.11

MMP-28 Interactions			
Ligand	Receptor	Type	Distance (Å)
Ar1	His 240	Pi stacking	3.92
O1	Leu 205	Hydrogen bond	2.01
O1	Ala 206	Hydrogen bond	2.41
O2	Ala 206	Hydrogen bond	2.57
O2	Glu 241	Hydrogen bond	2.34
O2	Zn 522	Metal coordination	2.35
O3	Zn 522	Metal coordination	2.19
O3	Zn 522	Salt bridge	2.19

Further, docking simulation confirmed that BIC formed a metal coordination complex with Zn^++^ in the catalytic domain of all tested MMPs, validating that all INSTIs possess chemical abilities to inhibits MMPs activities by chelating Zn++ at the catalytic domain. Metal coordination of BIC with Zn++ occurred at Zn 471, 166, 479, 269, 469, 709, 478, 490, 472, 473, 584, 671, 609, 605, 510, 485, 571, 392, 647, 564, 263, 515, and 522 receptors of MMP-1, 2, 3, 7, 8, 9, 10, 11, 12, 13, 14, 15, 16, 17, 19, 20, 21, 23, 24, 25, 26, 27, and 28, respectively. Like CAB, BIC also formed salt bridges with Zn++ of all tested MMPs. Along with Zn++, BIC was found to form salt bridge interactions with glutamate amino acids of all tested MMPs except MMP-2, 13, 15, 24 and 26. Cation pi interactions occurred only at phenylalanine 241 of MMP-24. Pi stacking interactions occurred with histidine amino acids of MMP-1, 9, 14, 17, 24, 26, 27, and 28; tyrosine amino acids of MMP-3, 10, 16, and 19, phenylalanine amino acids of MMP-12 and 24; tryptophan amino acid of MMP-26. Hydrogen bonding of BIC was observed with all MMPs except MMP-2, 19, 28. Like DTG and CAB, BIC was found to form hydrogen bonds with asparagine, histidine, leucine, alanine, proline, glutamate, valine, phenylalanine, glycine, and arginine amino acid residues. The distances of all the receptor-ligand bonds are shown in the respective BIC-MMP interaction table (Table 3). Docking simulation of BIC into individual MMP showed binding energy of −14.55, −10.972, −15.082, −11.385, −15.771, −13.415, −16.021, −12.143, −13.482, −12.73, −14.539, −8.877, −14.625, −12.409, −11.695, −11.923, −12.698, −12.25, −10.181, −11.716, −11.971, −12.54, and −10.823 kcal/mol for MMP-1 to −28, respectively (Table 4). Observed high binding energies and docking interactions (Table 1; Table 4) confirmed that BIC is a broad-spectrum MMPs inhibitor.

**TABLE 3:** Bictegravir (BIC)- Matrix metalloproteinases (MMPs) Interactions.

MMP-1 Interactions			
Ligand	Receptor	Type	Distance (Å)
Ar1	His 183	Pi stacking	4.4
O1	Asn 179	Hydrogen bond	1.99
O1	His 183	Hydrogen bond	2.64
O3	Zn 471	Metal coordination	1.99
O3	Zn 471	Salt bridge	1.99
O4	Zn 471	Metal coordination	2.2
O4	Glu 219	Salt bridge	4.4
O5	Leu 181	Hydrogen bond	2.38

MMP-2 Interactions			
Ligand	Receptor	Type	Distance (Å)
O2	Zn 166	Metal coordination	2.24
O3	Zn 166	Metal coordination	2.05
O3	Zn 166	Salt bridge	2.05

MMP-3 Interactions			
Ligand	Receptor	Type	Distance (Å)
Ar1	Tyr 185	Pi stacking	5.35
O3	Zn 479	Metal coordination	2.09
O3	Zn 479	Salt bridge	2.09
N2	Glu 219	Salt bridge	4.64
O4	Zn 479	Metal coordination	2.13
O5	Leu 181	Hydrogen bond	2.25

MMP-7 Interactions			
Ligand	Receptor	Type	Distance (Å)
O1	Ala 179	Hydrogen bond	2.06
N2	Glu 215	Salt bridge	4.86
O3	Zn 269	Metal coordination	2.04
O3	Zn 269	Salt bridge	2.04
O4	Zn 269	Metal coordination	2.07

MMP-8 Interactions			
Ligand	Receptor	Type	Distance (Å)
O1	Asn 170	Hydrogen bond	2.19
O3	Zn 469	Metal coordination	2.04
O3	Zn 469	Salt bridge	2.04
N2	Glu 218	Salt bridge	4.71
O4	Zn 469	Metal coordination	2.29
O5	Leu 180	Hydrogen bond	2.3

MMP-9 Interactions			
Ligand	Receptor	Type	Distance (Å)
Ar1	His 401	Pi stacking	3.84
O2	Pro 421	Hydrogen bond	2.28
O2	Zn 709	Metal coordination	2.29
O3	Zn 709	Metal coordination	2.01
O3	Zn 709	Salt bridge	2.01
N2	Glu 402	Salt bridge	4.3

MMP-10 Interactions			
Ligand	Receptor	Type	Distance (Å)
Ar1	Tyr 184	Pi stacking	5.01
O1	Ala 183	Hydrogen bond	2.03
N2	Glu 218	Salt bridge	4.58
O3	Zn 478	Metal coordination	1.96
O3	Zn 478	Salt bridge	1.96
O4	Zn 478	Metal coordination	2.11
O5	Leu 180	Hydrogen bond	1.96

MMP-11 Interactions			
Ligand	Receptor	Type	Distance (Å)
O1	Ala 180	Hydrogen bond	1.91
O3	Zn 490	Metal coordination	2.26
O3	Zn 490	Salt bridge	2.26
N2	Glu 216	Salt bridge	4.69
O4	Zn 490	Metal coordination	2.21
O5	Leu 177	Hydrogen bond	1.9

MMP-12 Interactions			
Ligand	Receptor	Type	Distance (Å)
Ar1	Phe 171	Pi stacking	5.1
O1	His 172	Hydrogen bond	2.01
O3	Zn 472	Metal coordination	2.03
O3	Zn 472	Salt bridge	2.03
N2	Glu 219	Salt bridge	4.44
O4	Zn 472	Metal coordination	2.02
O5	Leu 181	Hydrogen bond	1.91

MMP-13 Interactions			
Ligand	Receptor	Type	Distance (Å)
O1	Leu 185	Hydrogen bond	1.92
O1	Ala 186	Hydrogen bond	1.85
O2	Glu 223	Hydrogen bond	1.98
O3	Zn 473	Metal coordination	1.99
O3	Zn 473	Salt bridge	1.99

MMP-14 Interactions			
Ligand	Receptor	Type	Distance (Å)
Ar1	His 239	Pi stacking	3.69
O1	Leu 199	Hydrogen bond	2.61
O2	Zn 584	Metal coordination	2.24
O3	Zn 584	Metal coordination	2.07
O3	Zn 584	Salt bridge	2.07
N1	Glu 240	Hydrogen bond	2.64
N2	Glu 240	Salt bridge	3.61

MMP-15 Interactions			
Ligand	Receptor	Type	Distance (Å)
O1	Leu 219	Hydrogen bond	1.92
O2	Zn 671	Metal coordination	2.19
O3	Zn 671	Metal coordination	2.18
O3	Zn 671	Salt bridge	2.18

MMP-16 Interactions			
Ligand	Receptor	Type	Distance (Å)
Ar1	Tyr 210	Pi stacking	5.02
O3	Zn 609	Metal coordination	2.01
O3	Zn 609	Salt bridge	2.01
N2	Glu 247	Salt bridge	4.06
O4	Zn 609	Metal coordination	2.18
O5	Leu 206	Hydrogen bond	2.05

MMP-17 Interactions			
Ligand	Receptor	Type	Distance (Å)
Ar1	His 209	Pi stacking	4.62
O3	Zn 605	Metal coordination	2.09
O3	Zn 605	Salt bridge	2.09
N2	Glu 249	Salt bridge	4.63
O4	Zn 605	Metal coordination	2.02
O5	Val 207	Hydrogen bond	2.33

MMP-19 Interactions			
Ligand	Receptor	Type	Distance (Å)
O2	Zn 510	Metal coordination	2.03
Ar2	Tyr 234	Pi stacking	4.39
O3	Zn 510	Metal coordination	2.04
O3	Zn 510	Salt bridge	2.04
N2	Glu 213	Salt bridge	3.79

MMP-20 Interactions			
Ligand	Receptor	Type	Distance (Å)
O3	Zn 485	Metal coordination	1.99
O3	Zn 485	Salt bridge	1.99
N2	Glu 227	Salt bridge	4.19
O4	Zn 485	Metal coordination	2.07
O5	Leu 189	Hydrogen bond	2.27

MMP-21 Interactions			
Ligand	Receptor	Type	Distance (Å)
N1	Glu 248	Hydrogen bond	1.93
O3	Zn 571	Metal coordination	2
O3	Zn 571	Salt bridge	2.2
N2	Glu 248	Salt bridge	3.33
N2	Glu 284	Salt bridge	4.15
O4	Zn 571	Metal coordination	2.2
O5	Phe 249	Hydrogen bond	2.23

MMP-23 Interactions			
Ligand	Receptor	Type	Distance (Å)
N1	Glu 167	Hydrogen bond	1.99
O3	Zn 392	Metal coordination	2.15
O3	Zn 392	Salt bridge	2.15
N2	Glu 212	Salt bridge	4.47
O4	Zn 392	Metal coordination	2.24
O5	Leu 168	Hydrogen bond	1.87

MMP-24 Interactions			
Ligand	Receptor	Type	Distance (Å)
Ar1	His 282	Pi stacking	4.35
O1	Leu 242	Hydrogen bond	2.14
O1	Ala 243	Hydrogen bond	2.64
O2	Glu 283	Hydrogen bond	2.14
O2	Zn 647	Metal coordination	2.34
Ar2	Phe 241	Pi stacking	3.99
O3	Zn 647	Metal coordination	2.08
O3	Zn 647	Salt bridge	2.08
N2	Phe 241	Cation pi	3.62

MMP-25 Interactions			
Ligand	Receptor	Type	Distance (Å)
O3	Zn 564	Metal coordination	2.14
O3	Zn 564	Salt bridge	2.14
N2	Glu 234	Salt bridge	4.33
O4	Zn 564	Metal coordination	2.05
O5	Leu 192	Hydrogen bond	2.35

MMP-26 Interactions			
Ligand	Receptor	Type	Distance (Å)
Ar1	His 208	Pi stacking	4.05
Ar1	Trp 231	Pi stacking	5.45
O1	Gly 172	Hydrogen bond	1.94
O2	Glu 209	Hydrogen bond	1.94
O2	Zn 263	Metal coordination	2.29
O3	Zn 263	Metal coordination	1.96
O3	Zn 263	Salt bridge	1.96

MMP-27 Interactions			
Ligand	Receptor	Type	Distance (Å)
Ar1	His 181	Pi stacking	3.71
O1	Arg 170	Hydrogen bond	1.95
O3	Zn 515	Metal coordination	2.14
O3	Zn 515	Salt bridge	2.14
N2	Glu 217	Salt bridge	4.14
O4	Zn 515	Metal coordination	2.28
O5	Leu 179	Hydrogen bond	2.13

MMP-28 Interactions			
Ligand	Receptor	Type	Distance (Å)
Ar1	His 240	Pi stacking	4.07
O2	Zn 522	Metal coordination	2.14
O3	Zn 522	Metal coordination	1.92
O3	Zn 522	Salt bridge	1.92
N2	Glu 241	Salt bridge	4.79

**TABLE 4:** Binding energies for each MMP with DTG, CAB or BIC.

Structure	DTG [Energy (kcal/mol)]	CAB [Energy (kcal/mol)]	BIC [Energy (kcal/mol)]
MMP-1	−6.032	−14.251	−14.55
MMP-2	−6.450	−8.588	−10.972
MMP-3	−6.253	−15.222	−15.082
MMP-7	−7.243	−12.305	−11.385
MMP-8	−8.330	−14.337	−15.771
MMP-9	−9.430	−14.222	−13.415
MMP-10	−6.686	−14.592	−16.021
MMP-11	−6.210	−10.352	−12.143
MMP-12	−6.713	−12.19	−13.482
MMP-13	−6.461	−11.798	−12.73
MMP-14	−9.040	−12.983	−14.539
MMP-15	−5.885	−9.632	−8.877
MMP-16	−7.325	−12.249	−14.625
MMP-17	−6.810	−11.666	−12.409
MMP-19	−7.130	−12.718	−11.695
MMP-20	−6.097	−12.413	−11.923
MMP-21	−6.179	−14.389	−12.698
MMP-23	−6.213	−13.109	−12.25
MMP-24	−6.917	−15.339	−10.181
MMP-25	−6.865	−11.62	−11.716
MMP-26	−7.198	−12.381	−11.971
MMP-27	−7.427	−12.614	−12.54
MMP-28	−6.012	−10.11	−10.823

For comparative evaluations, docking simulations were also performed using the known broad-spectrum MMPs inhibitor DOX. DOX is the only US Food and Drug Administration (FDA)-approved broad-spectrum MMPs inhibitor. These were performed against five MMPs. These included MMP-2, 8, 9, 14, and 19. These MMPs were selected as INSTIs have higher binding energies with these enzymes compared to others and each enzyme represented different class of the MMP family. DOX was found to form metal coordination with Zn++ for all tested MMPs. These metal co-ordinations occurred at Zn 166, 469, 709, 584, and 510 receptors of MMP-2, 8, 9, 14, and 19 respectively. Pi stacking interactions occurred with histidine amino acids of MMP-2 and 14. Hydrogen bonding of DOX occurred with all five MMPs tested. DOX was found to form hydrogen bonds with glutamate, alanine, aspartic acid, tyrosine, asparagine, serine, and proline amino acid residues. The distances of receptor-ligand bonds are shown in the DOX-MMP interaction table (Supplementary Table S1). Further, docking simulations of DOX into individual MMPs showed binding energies of −6.595, −7.024, −7.658, −7.114, and −6.488 kcal/mol for MMP-2, 8, 9, 14, and 19 respectively. In comparison to DOX, all three INSTIs (DTG, CAB and BIC) showed higher binding energies with each tested MMP. Interestingly, both, CAB and BIC, showed significantly higher energies compared to DOX and DTG, suggesting CAB or BIC may have comparatively stronger inhibition effect on MMPs (Supplementary Table S2).

**FIGURE 1:**
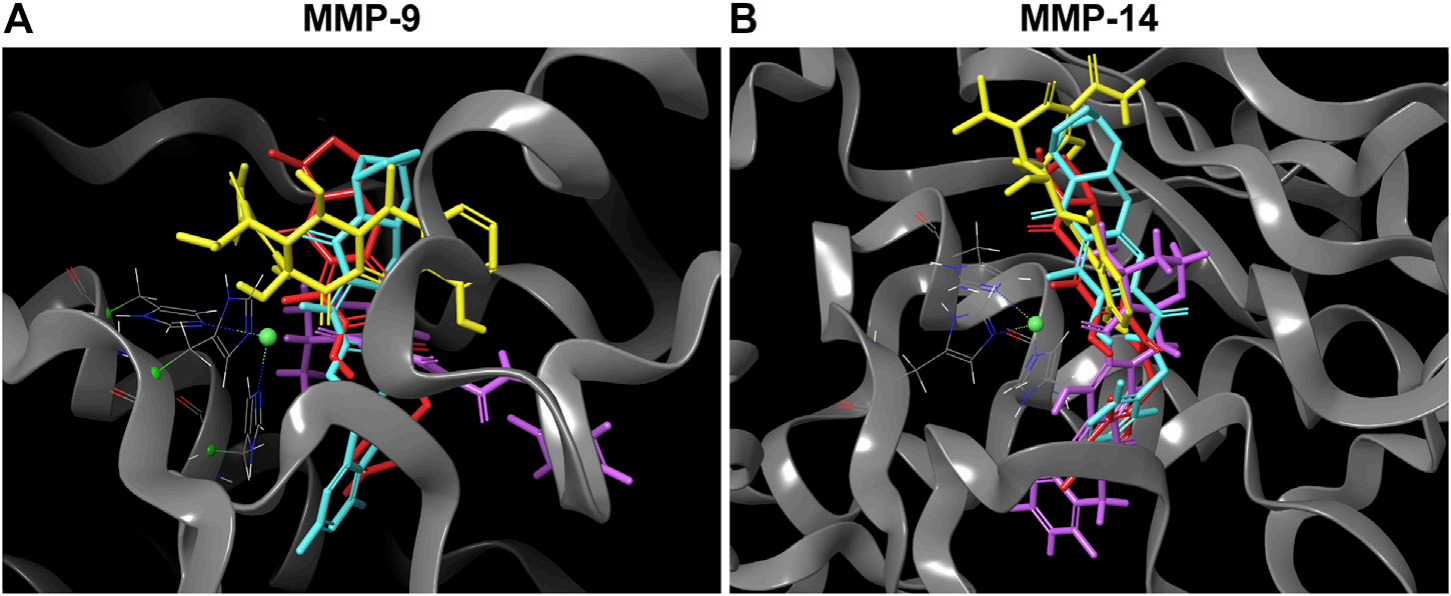
Superior affinity of DTG, CAB, BIC compared to DOX at MMPs catalytic binding site. **(A and B)** 3D representative images of overlapping molecular docking complexes of DTG, CAB, BIC, and DOX on MMP-9 or -14 catalytic domain containing Zn++ (green ball) are shown in ribbon (gray color) format. The color scheme utilized for drugs is as follows: DTG - Magenta; CAB - Red; BIC - Light blue; and DOX - Yellow.

The lower binding energy of DOX compared to INSTIs can be explained by its fit within the catalytic binding site. An overlay of the docked DTG, CAB, BIC and DOX on catalytic domain of MMP-9 and -14 showed that DOX (yellow color) has more solvent exposed area than DTG (magenta color), BIC (light blue color) and CAB (red color) (Figure 1). Further, solvent accessible surface area (SASA) calculations confirmed the higher solvent exposure of DOX compared to any of the INSTI (Supplementary Table S3). The higher SASA values of docking complex indicate that the DOX is interacting at lesser extent with MMP’s structural binding site and has a higher affinity to form bonds with the solvent compared to INSTIs. These data confirmed that INSTIs fit the MMP binding pocket with greater efficiency than DOX.

### INSTIs-induced inhibition of MMPs activities

**FIGURE 2:**
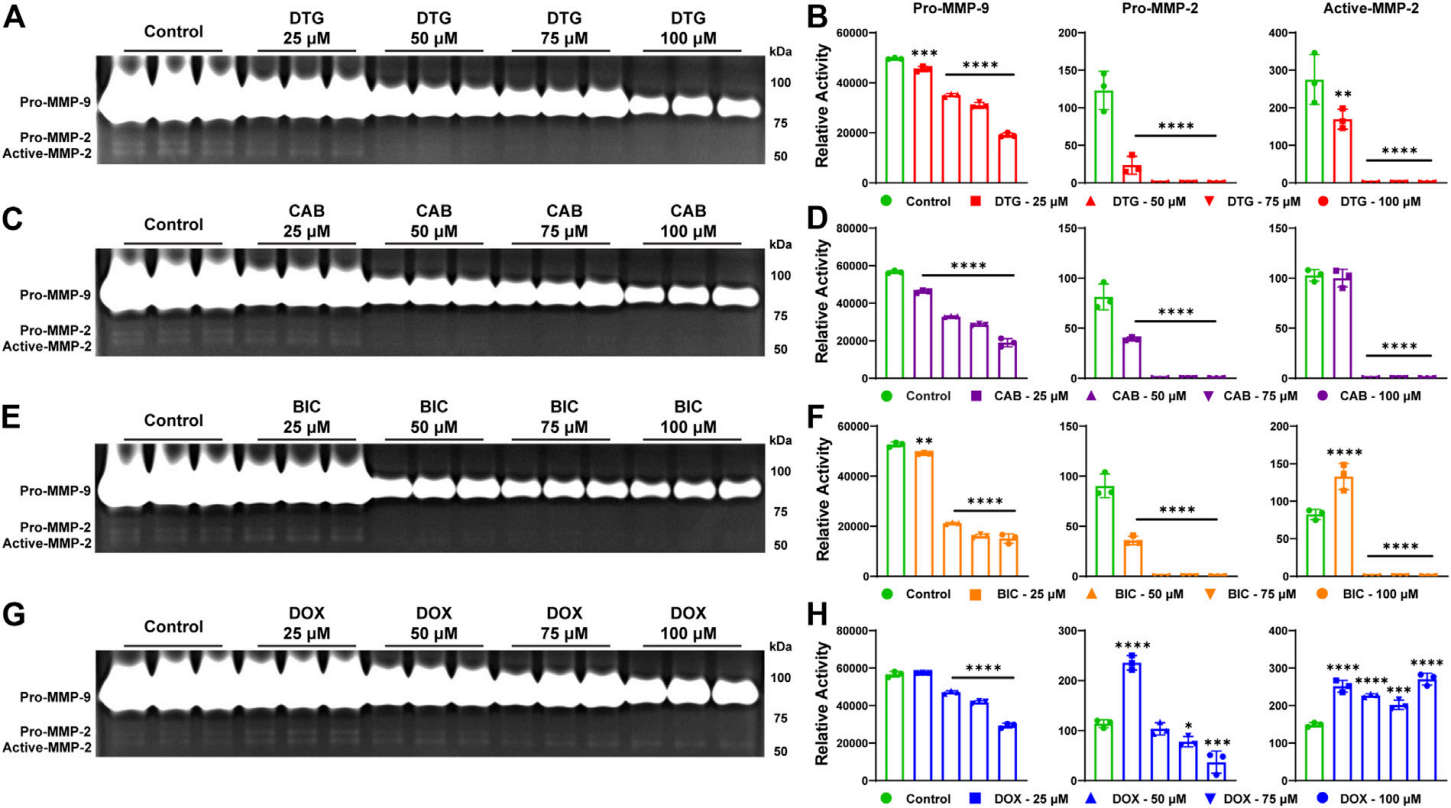
Inhibition of MMPs by INSTIs. **(A, C, E, and G)** Gelatin zymogram. Activity of MMP-2 and MMP-9 was evaluated in serum-free medium of THP-1 cells following treatment with DTG, CAB, BIC or DOX (25, 50, 75 or 100 µM). Vehicle treated cells were used as controls. **(B, D, F, and H)** Relative activity of MMP-9 or -2 was measured following treatment with DTG, CAB, BIC or DOX. A one-way ANOVA followed by Dunnett’s test was used to compare activity of individual MMP between each treatment concentration of individual drug and respective control (*p < 0.05, **p < 0.01, ***p < 0.001, ***p < 0.0001). Data are expressed as the mean ± SEM, N = 3 biological replicates. Experiments were repeated independently three times with equivalent results.

To affirm that inhibition of MMPs activity is an INSTI class effect, gelatin zymography, a commonly used assay to study MMPs activity and their inhibitors, was performed. For gelatin zymography, cell culture of THP-1 cells was utilized. Cells were treated with phorbol-12-myristate-13-acetate (PMA) to induce differentiation of THP-1 cells into macrophage like cells and to promote MMPs secretion. Herein, PMA-stimulated THP-1 cells were treated with escalating concentrations (25, 50, 75 or 100 µM) of DTG, CAB or BIC for 24 h in serum-free culture medium. Further, to validate the outcome, DOX was utilized as an MMP inhibitor control and the same treatment conditions were employed. To determine the proteolytic activity of MMP-2 and -9 (gelatinases), equal amount of protein (3 µg) from cell culture medium was loaded on SDS-PAGE containing gelatin. Gel area digested by both MMPs was visualized using Coomassie blue stain (Figures 2A, C, E). A decrease in activity of MMP-2 and -9 was observed following treatment with all three INSTIs compared to vehicle-treated controls on gelatin zymogram (Figures 2A, C, E). Relative activity of the pro forms of MMP-2 and MMP-9 was significantly decreased in a concentration-dependent manner at each tested concentration after treatment with DTG, CAB or BIC compared to controls (Figures 2B, D, F). Relative activity of the active form of MMP-2 was significantly reduced at all concentrations of DTG. However, for CAB and BIC, relative activity of the active form of MMP-2 was significantly reduced at 75 or 100 µM. Interestingly, there was a significant increase in relative activity of the active form of MMP-2 at 25 µM BIC compared to controls. Further, DOX treatment, showed a significant decrease in relative activity of pro-form of MMP-9 in a concentration dependent manner (Figures 2G, H). However, variable inhibition of MMP-2 was observed after DOX treatment (Figures 2G, H). Relative activity of the pro form of MMP-2 was significantly increased at 25 µM DOX, but significantly decreased in a concentration-dependent manner at 75 and 100 µM concentrations. Relative activity of the active form of MMP-2 was significantly increased at all treatment concentrations of DOX. When comparing DOX and INSTIs at the same treatment concentration, DTG, CAB, and BIC showed higher MMP inhibition compared to DOX (Supplementary Figure S1A–H). Overall, gelatin zymography results confirmed that inhibition of MMPs activities is an INSTI class effect. These results demonstrated that INSTIs inhibit MMP at a higher degree than the known broad-spectrum MMP inhibitor, DOX.

## Discussion

The risk of pre- or post-natal neurodevelopmental deficits due to gestational exposure to ARVs remains possible (Hill et al., 2018; Cassidy et al., 2019; Zash et al., 2019; Crowell et al., 2020; Williams et al., 2020). Works outlined in this report provide unique insights into the underlying mechanisms linked to such adverse events. Recently, clinical and pre-clinical studies reported a potential association between DTG usage at the time of conception and NTDs (Hill et al., 2018; Raesima et al., 2019; Zash et al., 2019; Kreitchmann et al., 2021) and postnatal neurological abnormalities(Crowell et al., 2020). Due to mass usage of DTG-based regimens worldwide, reports highlighted the need to find an underlying mechanism of potential DTG-related adverse neurodevelopmental outcomes. With the introduction of new potent ARVs from the INSTI class to treatment regimens, it is essential to establish if such mechanism can be linked to other ARVs from the INSTI class. Herein, we show that INSTIs including DTG, CAB, and BIC possess chemical abilities to interact with Zn++ at the catalytic domain of all twenty-three MMPs observed in humans and thus, can be classified as broad-spectrum MMPs inhibitors. Such secondary mechanism of MMPs inhibition introduces potential for adverse effects, especially during critical periods of fetal brain development.

All ARVs from the INSTI class possess metal-binding pharmacophore, MBP in their chemical structure. This chemical property enables INSTIs to interact with active metal ion (Mg++) sites in the HIV-1 integrase enzyme to block its action of insertion of the viral genome into the host cellular DNA (Smith et al., 2021). With such inherent metal chelating chemical property, INSTIs have potential to interact with other metalloenzymes that are critical for normal cellular functions such as cell proliferation, differentiation, cell signaling, protein cleavage, *etc.* MMPs are well recognized Zn++ dependent metalloenzymes (Ethell and Ethell, 2007; Page-McCaw et al., 2007; Agrawal et al., 2008; van Hinsbergh and Koolwijk, 2008; Loffek et al., 2011; Fujioka et al., 2012; Reinhard et al., 2015; Rempe et al., 2016; Small and Crawford, 2016; De Stefano and Herrero, 2017; Shinotsuka et al., 2018; Kanda et al., 2019). The active site of these enzymes is highly conserved, and comprised of three histidine residues that are bound to the catalytic zinc (Laronha and Caldeira, 2020). Dysregulation of activities of MMPs through chelation of Zn++ can cause detrimental effects on structural and functional development of the CNS. The chemical property of INSTIs to chelate divalent cations enables them to engage with Zn++ in the catalytic domain of all twenty-three human MMPs. Our comprehensive molecular docking assessments confirmed that inhibition of MMPs activity is an INSTI class effect. Notably, interaction of individual DTG, CAB or BIC with each MMP was variable with different binding energy. Thus, studies evaluating drug-induced inhibitions of individual MMPs under biological conditions is needed in the future to identify the susceptibility of individual MMP enzymes under normal and genetic polymorphism conditions.

The role of MMPs in normal neural development is of critical importance. MMPs expression is at high levels during early CNS development and decreases into adulthood (Vaillant et al., 1999; Ayoub et al., 2005; Ulrich et al., 2005; Larsen et al., 2006; Bednarek et al., 2009; Aujla and Huntley, 2014; Reinhard et al., 2015). Due to their proteolytic activities, MMPs are ubiquitously expressed during neural development and their expression has been majorly studied in hippocampus, cortex and cerebellum (Fujioka et al., 2012; Reinhard et al., 2015; Small and Crawford, 2016; Beroun et al., 2019). The principal function of MMPs is to degrade extracellular matrix components (Lukes et al., 1999). However, it is well recognized that MMPs functions are essential for the regulation of several neurodevelopmental processes including neurogenesis, neurite outgrowth, migration of newly born neurons, myelination, axonal guidance, synaptic plasticity and angiogenesis (Fujioka et al., 2012; Reinhard et al., 2015; Small and Crawford, 2016). Dysregulation of MMPs activities during critical periods of fetal brain development during gestation could significantly affect these processes, resulting in adverse neurodevelopmental outcomes (Fujioka et al., 2012; Reinhard et al., 2015; Small and Crawford, 2016). Notably, previously we observed that DTG inhibits MMPs activities in rodent embryo brain during gestation leading to neuroinflammation and neuronal injury in the CNS of mice pups during postnatal assessments (Bade et al., 2021). This study identified DTG-induced inhibition of MMPs activities as a neurotoxicity biomarker. However, the previous study was proof of concept and mainly focused on DTG, but comprehensive docking assessments for consideration of each of MMP was missing. The current study confirmed that all INSTIs possess abilities to inhibit MMPs activities. Therefore, drug-induced differences in MMP activities or MMP expression levels could serve as a biomarker for INSTI-associated neurodevelopmental impairments. In addition to pregnancy outcomes, INSTIs also have been recognized to be associated with neuropsychiatric adverse events (NPAEs) in adults (Yombi, 2018; Amusan et al., 2020; Senneker and Tseng, 2021) and clinically significant weight-gain, especially in females (NAMSAL ANRS 12313 Study Group et al., 2019; Venter et al., 2019; Bourgi et al., 2020; Caniglia et al., 2020; Sax et al., 2020). Impaired MMPs activity, expression and related cellular pathways have been identified as biomarkers in both disorders (Vandenbroucke and Libert, 2014; Jaoude and Koh, 2016; Shinotsuka et al., 2018; Beroun et al., 2019; Ruiz-Ojeda et al., 2019; Gorwood et al., 2020; Li et al., 2020). Further, due to critical functions of MMPs, their dysregulation has also served as a biomarker for several types of tumors and atherosclerosis (Goncalves et al., 2015; Huang, 2018). Thus, this work provides a potential mechanistic biomarker for neurodevelopmental assessments following *in utero* exposure to ART regimens with an INSTI component.

Identifying the roles of the MMPs and impact of their independent or broad-spectrum inhibition in physiological or pathological conditions is complex. This has reflected in cessation of clinical trials of more than fifty broad-spectrum MMP inhibitors due to adverse events following prolonged treatment (Vandenbroucke and Libert, 2014). Thus, an understanding of the inhibitory effect of INSTIs against each MMP enzyme is essential to define the mechanism linked to neurodevelopment. The INSTIs utilized for testing in this study DTG, CAB and BIC showed strong binding energy with the catalytic domains of all twenty-three MMPs. Moreover, comparison assessments against DOX, a clinically used broad-spectrum inhibitor of MMPs, confirmed the high potency of DTG, CAB or BIC against MMPs. For example, binding energies (kcal/mol) with the catalytic domain of MMP-9 were −9.430 (DTG), −14.222 (CAB), and −13.415 (BIC) against −7.658 (DOX). These molecular docking assessment differences against MMP-9 were further apparent on the confirmatory gelatin zymography biological tests. Interestingly, CAB and BIC exhibited higher binding energies for all tested MMPs compared to DTG, suggesting that these newer INSTIs may be more potent MMPs inhibitors. Such observations will need biological validations along with determination of half maximal inhibitory concentration (IC_50_) values of each INSTI against individual enzymes in the future.

Of the twenty-three human MMPs, few MMPs are well characterized for their role during neurodevelopment. MMP-2 and MMP-9 have been studied extensively and are shown to be essential for the neuronal development, migration, axonal guidance and synaptic plasticity (Ethell and Ethell, 2007; Page-McCaw et al., 2007; Agrawal et al., 2008; Fujioka et al., 2012; Reinhard et al., 2015; Small and Crawford, 2016; De Stefano and Herrero, 2017). Widespread expression of MMP-3 has been identified in neurons in the brain and spinal cord of rodents during critical timepoints of axonal outgrowth (Van Hove et al., 2012a). Moreover, MMP-24 is also expressed in neurons in the brain and spinal cord during development, signifying its role in neuronal development, and MMP-2 and -14 are involved in angiogenesis and in establishment and/or maintenance of the blood-brain barrier (BBB) (Girolamo et al., 2004; Lehti et al., 2005; Lehti et al., 2009; Ikonomidou, 2014; Rempe et al., 2016; Kanda et al., 2019). Knock out or knockdown models of the mentioned MMPs have proved that deficiency in these MMPs can affect neurodevelopmental processes (Oh et al., 2004; Luo, 2005; Van Hove et al., 2012b; Kanda et al., 2019). Interestingly, MMPs are expressed abundantly in neural stem cells (Frolichsthal-Schoeller et al., 1999). Inhibition of MMPs activity by synthetic inhibitors was shown to reduce proliferation and differentiation of neural stem cells (Szymczak et al., 2010; Wojcik-Stanaszek et al., 2011). Thus, identifying the impact of INSTI-induced inhibition of MMPs activities on neurodevelopment and unravelling genetic susceptibility increasing the severity of adverse effects will be critical.

Clinical and pre-clinical studies showed high levels of transplacental transfer of INSTI drugs (Schalkwijk et al., 2016; Mulligan et al., 2018; Mandelbrot et al., 2019; Waitt et al., 2019; Bollen et al., 2021; Bukkems et al., 2021). Studies of DTG have found high placental transfer of DTG from mother to fetus with median cord blood to maternal blood drug level ratios from 1.21 up to 1.29 (Schalkwijk et al., 2016; Mulligan et al., 2018; Mandelbrot et al., 2019; Waitt et al., 2019; Bollen et al., 2021). Further, DTG was also found to accumulate in the fetus with noted prolonged elimination of drug from infants after birth (Mulligan et al., 2018; Waitt et al., 2019). Although few studies have addressed placental transfer of CAB and BIC, evidence does suggest that these INSTIs also cross the placental barrier (Pencole et al., 2020; Bukkems et al., 2021; Le et al., 2022). Our previous work investigated pharmacokinetic (PK) and biodistribution (BD) of DTG during pregnancy in mice and confirmed that DTG levels are detectable in brain tissues of embryos following daily oral administration at supratherapeutic dosage (Bade et al., 2021). Our work validated clinical reports of high placental transfer of DTG and was the first to show drug levels in the fetal developing brain during gestation. Such transplacental transfer of DTG indicated that direct exposure of the embryo brain to DTG during critical periods of development could have an adverse impact on neurodevelopment. Therefore, understanding the PK and BD profiles of new INSTIs during pregnancy and their effects on neurodevelopmental processes is needed for better mechanistic assessments.

It is acknowledged that despite the occurrence of birth defects has been a concern, both the United States Department of Health and Human Services (DHHS) and World Health Organization recommend DTG as a preferred first-line ARV during pregnancy (The U.S. Department of Health and Human Services, 2015; World Health Organization (WHO), 2019a). This decision was based on risk benefit ratios offered by DTG as an ARV compared to rate of associated risk. These included fewer mother-to-child HIV-1 transmission and maternal deaths, and cost-effective (Dugdale et al., 2019; Phillips et al., 2020). Moreover, DTG’s high genetic barrier to drug resistance would address the critical problem of rising pretreatment resistance (PDR) to non-nucleoside reverse transcriptase inhibitors (NNRTIs) in RLCs, especially in women (World Health Organization (WHO), 2019a; World Health Organization (WHO), 2019b). Moreover, most updated data from Tsepamo study (Botswana) reported declined rate of birth defects and was comparative between DTG and other ARVs at the time of conception in late breaking abstract at the 24th International AIDS Conference, 2022. Nonetheless, assessment of birth defects in Botswana is an ongoing study and recommended guidelines were based on higher benefits offered by DTG. Yet, risk of long-term neurodevelopmental deficits persists. Particularly, there is a research gap of known adverse events reflecting DTG-associated long-term impact on postnatal neurodevelopment. Therefore, with large number of fetuses being exposed to DTG worldwide, continuous research efforts are critical to uncover any adverse effects of DTG exposures on pre- or post-natal neurodevelopment and elucidate underlying mechanism.

Although the current study provides evidence of an INSTI class effect on the inhibition of MMPs, it was limited to laboratory cell-based assessments. Future studies are necessary in order to affirm mechanistic links between altered MMP activities and adverse developmental outcomes following *in utero* INSTI exposures. Dose dependent effects of each INSTI on MMPs activities at different stages of neurodevelopment during gestation and early postnatal period need to be studied in animal models. This work would need to include detailed BD drug profiles within the fetal CNS and related MMPs activities. Moreover, with a metal chelating motif, INSTIs possess potential to inhibit other metalloenzymes required for fetal brain development such as Zn++ dependent a disintegrin and metalloproteinase (ADAM) family members (Jorissen et al., 2010; Vandenbroucke and Libert, 2014). Whether inhibition of these metalloenzymes, even at minimal extent, in addition to MMPs could augment the developmental adverse events needs consideration. Thus, comprehensive computational modeling against other metalloenzymes along with biological validations are critical in the future. Moreover, development of ultra-long acting nanoformulations of DTG and assessment of these as a safe drug delivery system for neuroprotective outcomes will be the focus our own future work. We hypothesize that neuroprotective effect would be the outcome of lower drug biodistribution in the embryo brain preventing MMPs inhibition. Such lower drug biodistribution in fetal brain while maintaining therapeutic drug levels in maternal blood is expected due to long-acting pharmacological properties of formulations and lower total drug administration compared to daily oral drug administration. For example, the 8-week cumulative dose of daily oral CAB (VOCABRIA) is 1,680 mg. Whereas, a 600 mg bi-monthly single intramuscular injection of LA-CAB (CABENUVA or APRETUDE) results in a 3-fold reduction in drug exposure with equivalent duration of action (US Food and Drug Administration (FDA), 2021; US Food and Drug Adminis tration (FDA), 2021). Importantly, scientific exchange between basic science mechanistic findings and the clinical assessment of INSTI-exposed children will be required in the future to provide cross-validation of scientific findings and rigorous assessments of neurodevelopment. Overall, it is timely to elucidate any potential ARV-induced secondary effects during pregnancy, in order to provide effective care for women and their fetuses. This study confirms that INSTIs are broad-spectrum MMPs inhibitors. As balanced regulation of MMP activities are crucial for neurodevelopment, the enzyme’s inhibition could underlie INSTI-related adverse neurodevelopmental outcomes.

## Data Availability

The original contributions presented in the study are included in the article/Supplementary Material, further inquiries can be directed to the corresponding author.
