# Supplementary material for: Inhibition of matrix metalloproteinases by HIV-1 integrase strand transfer inhibitors
Source: Front Toxicol. 2023 Feb 21;5:1113032. doi: 10.3389/ftox.2023.1113032 (PMC9988942; doi:10.3389/ftox.2023.1113032)
Supplement: Supplementary file 1 [file DataSheet1.docx]

**Inhibition of Matrix Metalloproteinases by HIV-1 Integrase Strand Transfer Inhibitors**

Emma G. Foster^1^, Nicholas Y. Palermo^2^, Yutong Liu^3^, Benson Edagwa^1^,

Howard E. Gendelman^1,4^, Aditya N. Bade^1^*

^1^Department of Pharmacology and Experimental Neuroscience, University of Nebraska Medical Center, Omaha, NE 68198, USA.

^2^Computational Chemistry Core, University of Nebraska Medical Center, Omaha, NE 68198, USA.

^3^Department of Radiology, University of Nebraska Medical Center, Omaha, NE 68198, USA.

^4^Department of Pharmaceutical Sciences, University of Nebraska Medical Center, Omaha, NE 68198, USA.

***Corresponding author:** Aditya N. Bade, Ph.D., Department of Pharmacology and Experimental Neuroscience, University of Nebraska Medical Center, Omaha, NE 68198-5800, USA; phone: 402-559-8295; fax: 402-559-7495; email: [aditya.bade@unmc.edu](mailto:aditya.bade@unmc.edu), ORCID: <https://orcid.org/0000-0003-2511-4461>

**Supplementary Material**

**Supplementary Figure 1**. **Comparison of MMPs inhibition by INSTIs and doxycycline.** (**A, C, E, G**) Gelatin zymogram. Activity of MMP-2 and MMP-9 was evaluated in serum-free medium of THP-1 cells following treatment with DTG, CAB, BIC or DOX (25, 50, 75 or 100 µM). Vehicle treated cells were used as controls. (**B, D, F, H**) Relative activity of MMP-9 or -2 was measured following treatment with DTG, CAB, BIC or DOX. A one-way ANOVA followed by Tukey's test was used to compare activity of individual MMP between different drugs at the same concentration compared to vehicle treated control (*P < 0.05, **P < 0.01, ***P < 0.001, ***P < 0.0001) and a positive-control for MMP inhibition, DOX (^$^P < 0.05, ^$$^P < 0.01, ^$$$^P < 0.001, ^$$$$^P < 0.0001). Data are expressed as the mean ± SEM, N = 3 biological replicates.


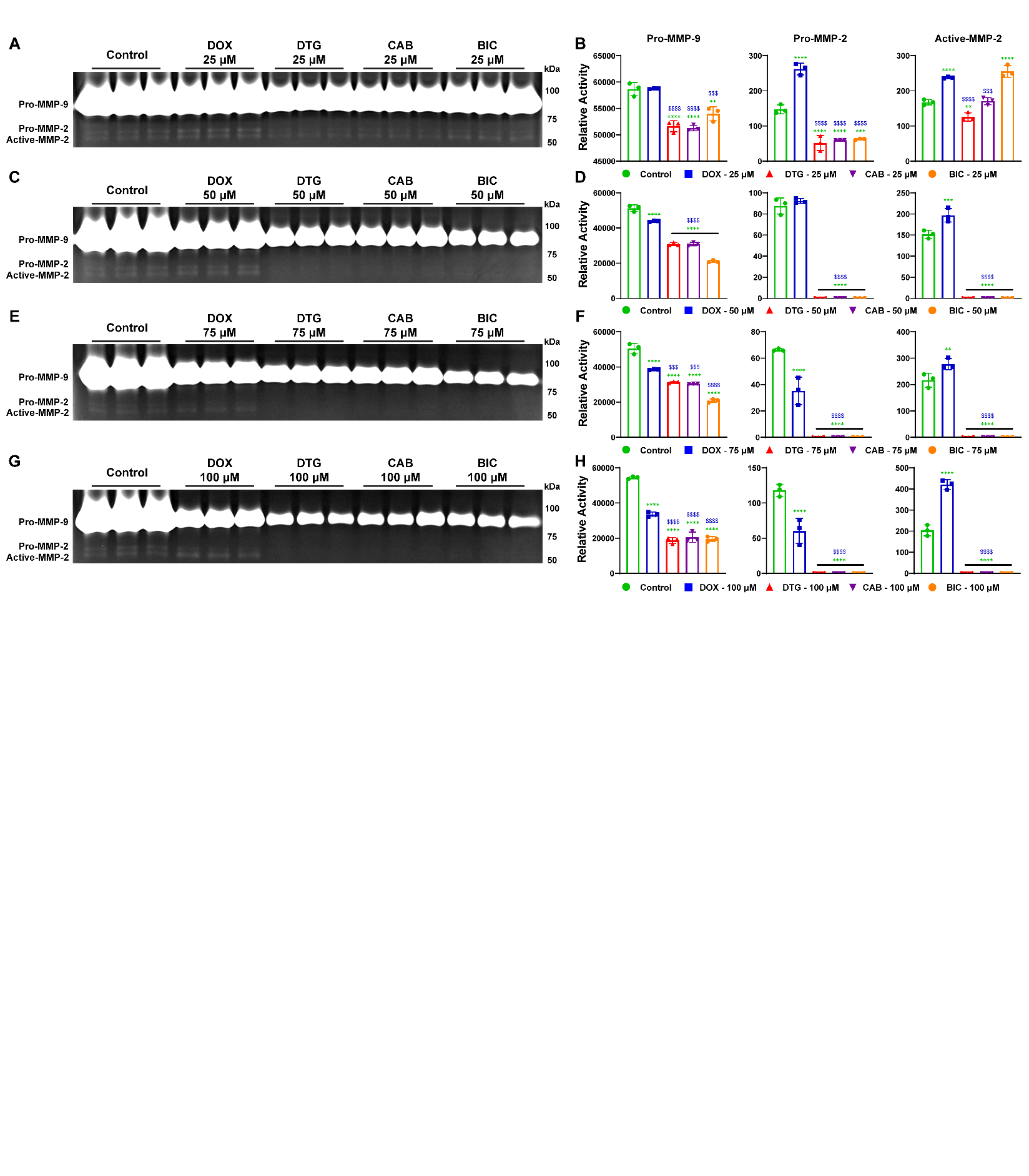


**Supplementary Table 1.** Doxycycline (DOX)- Matrix metalloproteinases (MMPs) Interactions

**Supplementary Table 2.** Comparative Assessment

**Supplementary Table 3**. Solvent accessible surface area (SASA) calculations
